# Microbial community structure of two freshwater sponges using Illumina MiSeq sequencing revealed high microbial diversity

**DOI:** 10.1186/s13568-016-0211-2

**Published:** 2016-06-14

**Authors:** Swapnil Gaikwad, Yogesh S. Shouche, Wasudev N. Gade

**Affiliations:** Department of Biotechnology, Savitribai Phule Pune University, Ganeshkhind, Pune, Maharashtra 411007 India; Microbial Culture Collection (MCC), First Floor, Central Tower, Sai Trinity Building Garware Circle, Sutarwadi, Pashan Pune, Maharashtra 411021 India

**Keywords:** Freshwater sponges, Sponge microbiota, Biotechnological potential, Next generation sequencing

## Abstract

Sponges are primitive metazoans that are known to harbour diverse and abundant microbes. All over the world attempts are being made to exploit these microbes for their biotechnological potential to produce, bioactive compounds and antimicrobial peptides. However, the majority of the studies are focussed on the marine sponges and studies on the freshwater sponges have been neglected so far. To increase our understanding of the microbial community structure of freshwater sponges, microbiota of two fresh water sponges namely, *Eunapius carteri* and *Corvospongilla lapidosa* is explored for the first time using Next Generation Sequencing (NGS) technology. Overall the microbial composition of these sponges comprises of 14 phyla and on an average, more than 2900 OTUs were obtained from *C. lapidosa* while *E. carteri* showed 980 OTUs which is higher than OTUs obtained in the marine sponges. Thus, our study showed that, fresh water sponges also posses highly diverse microbial community than previously thought and it is distinct from the marine sponge microbiota. The present study also revealed that microbial community structure of both the sponges is significantly different from each other and their respective water samples. In the present study, we have detected many bacterial lineages belonging to *Firmicutes, Actinobacteria, Proteobacteria, Planctomycetes*, etc. that are known to produce compounds of biotechnological importance. Overall, this study gives insight into the microbial composition of the freshwater sponges which is highly diverse and needs to be studied further to exploit their biotechnological capabilities.

**Electronic supplementary material:**

The online version of this article (doi:10.1186/s13568-016-0211-2) contains supplementary material, which is available to authorized users.

## Introduction

Sponges are one of the most primitive organisms that lack definitive tissue grade organisation. They (especially marine sponges) are being studied all over the world not only because of its ecological and evolutionary significance, but also due to their ability to produce bioactive compounds (Grozdanov and Hentschel 2007; Taylor et al. 2007; Hardoim and Costa 2014). The empirical evidences suggest that most of these compounds are produced by microbes associated with them (Anand et al. 2006). Due to the potential use of these bioactive compounds in the pharmaceutical industries, researchers have focussed on the understanding and exploration of the microbial composition of sponges (Patnayak and Sree 2005; Santos-Gandelman et al. 2014). In fact, studies in the past have suggested that almost 40–60 % cell mass of the sponges is composed of bacterial cells (Santos-Gandelman et al. 2014 and references mentioned therein). Studies have also shown that sponge associated microbes not only help in the biogeochemical cycles, but also plays an important role in utilising dissolved organic matter, thus helps in maintaining the benthic food web (de Goeij et al. 2008). Furthermore, microbial community structure of sponges is also thought to be associated with the morphological and metabolic features of the sponges (Schöttner et al. 2013).

However, most of the studies are restricted to the marine sponges as they are diverse and abundantly present compared to their freshwater counterparts (van Soest et al. 2012). The knowledge about the microbial composition of freshwater sponges is still lacking. It is presumed that existing symbionts could have been lost when freshwater sponges colonized their new habitat from the sea and this could be the reason for low bacterial diversity and abundance in the fresh water sponges (Taylor et al. 2007). Studies have suggested that even freshwater sponges are associated with diverse bacterial species (Gernert et al. 2005; Costa et al. 2012; Kaluzhnaya et al. 2012; Keller-Costa et al. 2014). Moreover, they are also known to produce bioactive compounds such as Syriacin (Rezanka et al. 2006) lipids, sterols, etc. (Dembitsky et al. 2003). Freshwater sponges are also known to show symbiotic association with insects; they also serve as a connecting link between pelagic and benthic food web (Skelton and Strand 2013). Thus like their marine counterparts freshwater sponges are also known to play crucial roles in maintaining the trophic ecology of lakes. The fresh water sponges are also considered as pollution indicator (Hill and Hill 2002) and are known to accumulate metals and other toxic compounds (de Barros et al. 2013). A study by Keller-Costa et al. (2014) showed, antimicrobial activity of various *Pseudomonas* species isolated from fresh water sponge, *Ephydatia fluviatilis* indicating their biotechnological potential. Thus, despite their important role in aquatic ecosystems, potential biotechnological and commercial use, studies on the exploration of the microbial community of the freshwater sponges are rare.

In the past, studies on the microbial composition of freshwater sponges were limited to the culture based identification (Parfenova et al. 2008), 16S rRNA clone library (Kaluzhnaya et al. 2012), DGGE analysis (Costa et al. 2012) and few studies focussed only on the specific bacterial groups (Keller-Costa et al. 2014; Kulakova et al. 2014). However, with the use of Next Generation Sequencing (NGS) technology, it is now possible to understand and explore the complex and highly diverse microbial community structure of freshwater sponges. This technique is being routinely used for the exploration of microbial communities of marine sponges (Lee et al. 2010; Cleary et al. 2013; Alex and Antunes 2015). In the present study, we aimed to explore and compare the microbial community structure of two taxonomically well characterised and commonly found fresh water sponges (*Eunapius carteri *and *Corvospongilla lapidosa*) based 16s rRNA gene sequencing using the Illumina Miseq platform. *E. carteri* (Family: Spongillidae) is a globular sponge and *C. lapidosa* (Family: Spongillidae) is an encrusting sponge.

## Materials and methods

### Sample collection

Samples of *E. carteri* and *C. lapidosa* were collected (in triplicates) from the permanent fresh water lake located at Talegaon Dabhade (labelled as TSPO) and Pashan (labelled as PSPO), respectively (collection details are given in Additional file 1: Table S1). Along with this, 350 ml of water samples in close proximity to the sponges were also collected from each collection site (TWAT and PWAT, respectively). At the collection site, we have also measured the ammonia, nitrite and phosphate content of the water and along with these parameters pH, temperature, total dissolved solids (TDS), conductivity and dissolved oxygen (DO) of the water were also measured using HANNA instruments (Italy) (Additional file 1: Table S2). Samples were immediately brought to the laboratory in separate sterile containers and washed with autoclaved distilled water to remove loosely attached debris and microbes and kept in vials containing absolute alcohol at −20 °C until further use. Water samples were immediately filtered through 0.2 µm pore sized hydrophilic polyvinylidene fluoride durapore membrane filter (Millipore, India) and filters were kept in −20 °C until further use.

### DNA isolation and sequencing

DNA from the sponge tissue and the membrane filters was isolated using the QIAamp DNA micro Kit (Netherlands) following manufacturer’s information. Isolated DNA was used for the exploration of microbial diversity using primers for V3–V4 region of 16S rRNA (Besemer et al. 2012). The high-throughput sequencing was performed using the Illumina Miseq platform by Xcelris Labs Ltd, Ahmedabad, India. The generated data were submitted to the MG-RAST under project id 4679561.3 to 4679572.3

### Data analysis

The adaptors and low quality sequences were removed using Trimmomatic v0.30 (Bolger et al. 2014). Paired ends were joined using QIIME (Caporaso et al. 2010). Obtained raw reads were quality filtered using Mothur (Schloss et al. 2009). In brief, sequences with read length of more than 400 bp, q value of more than 25 with no ambiguity and a homopolymer length of less than 6 bp were selected. Sequences were then used for microbial diversity analysis using QIIME. In brief, sequences were clustered into OTUs (Operational Taxonomic Unit) at 97 % similarity and OTU picking was done by an open reference method and chimera check was done using chimera-slayer. Taxonomic assignments were carried out using Greengene 13.8 database. Alpha diversity indices such as Chao1, observed_species, shannon and Good’s coverage were calculated using QIIME. Similarity percentage (SIMPER) analysis was done using PAST software (Hammer et al. 2001) to identify the OTUs that were responsible for the differences observed between the PSPO and the TSPO samples. This software was also used statistical analysis using T test. Shared OTUs (at genus level) between different samples was viewed using online tool Venny (http://www.bioinfogp.cnb.csic.es/tools/venny/index.html).

To determine the difference between the microbiota of freshwater sponges and marine sponges, the microbial composition of freshwater sponges from this study and marine sponges published by Schmitt et al. (2012) was compared based on NMDS plot. The sequences generated by Schmitt et al. (2012) were downloaded from Sequence Read Archive (SRA) under accession number SRP003545. To avoid biases in the analysis, these sequences were quality filtered using same criteria described above and compared with the sequences generated in this study.

### Statistical analysis

The differences in the microbial community structure within different individual of the samples and between the samples were determined using Statistical Analysis of Metagenomic Profiles (STAMP) (Parks and Beiko 2010) and the ANOVA using PAST (Hammer et al. 2001).

## Results

The ecological parameters measured for the collected water samples from both the localities were significantly different from each other (Additional file 1: Table S2). PWAT samples showed high nitrite and ammonia content while TWAT samples showed higher values for the phosphate content. Similarly, we also recorded higher values of pH, TDS, conductivity, DO and temperature for PWAT samples.

We obtained 1321,078 reads ranging from 52,206 to 172, 090 sequences per samples, after quality trimming, removal of singletons and chimeras (Table 1). The highest number of OTUs was observed in PSPO (2947 ± 269), PWAT (2629 ± 427), TWAT (2502 ± 84) followed by TSPO (987 ± 34).

**Table 1:** Summary of species richness estimators of the samples studied

No.	Samples	Good quality sequences	Chao1^a^	Observed species^a^	Shannon^a^	Simpson^a^	Goods coverage (%)^a^
1	PSPO1	144,561	5199	3215	7.68	0.98	97
2	PSPO2	168,769	5339	3218	6.84	0.93	97
3	PSPO3	150,149	3446	2408	7.7	0.98	98
4	PWAT1	86,401	2507	1840	7.43	0.98	99
5	PWAT2	102,448	4551	2740	7.55	0.98	97
6	PWAT3	88,352	5039	3308	8.19	0.99	97
7	TSPO1	67,388	1366	930	4.42	0.82	99
8	TSPO2	103,268	1471	982	4.95	0.88	99
9	TSPO3	100,068	1662	1049	5.27	0.91	99
10	TWAT1	172,090	4120	2435	7.15	0.96	98
11	TWAT2	85,384	3645	2403	7.16	0.96	98
12	TWAT3	52,206	3651	2670	7.91	0.99	98

The alpha diversity indices were calculated with 3 % distance cut-off

^a^These values were calculated after all samples were randomly sub-sampled with 52,206 sequences per samples as lowest number of sequences were found in TWAT3 sample

**Fig. 1:**
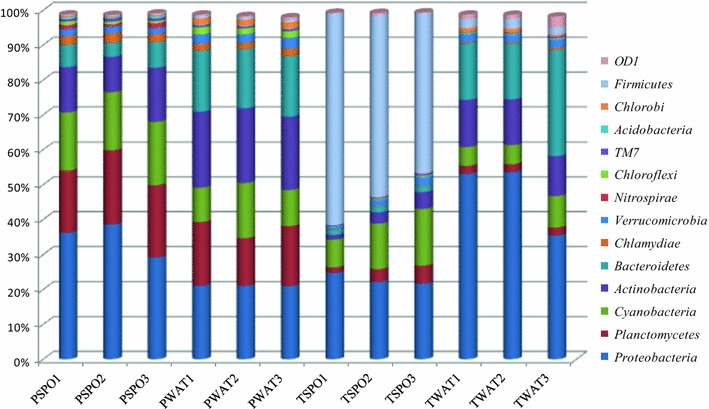
The relative abundance of different bacterial phyla in the samples studied. The abundance of the phyla is plotted on *Y axis*. The phyla showing <1 % relative sequence abundance are not shown here

We identified 14 most abundant phyla (represented by more than 1 % total bacterial sequences) in the freshwater sponges and its surrounding water (Fig. 1). In terms of relative abundance these phyla represented more than 99 % of the microbial community in the samples studied here. Our study showed significant differences between the microbial community structure of TSPO and PSPO samples (Additional file 1: Fig. S1). Sample TSPO was dominated by *Firmicutes* followed by *Proteobacteria*, *Cyanobacteria* while PSPO was dominated by *Proteobacteria* followed by *Planctomycetes, Cyanobacteria* and *Actinobacteria*. The water from the sampling sites of the sponges also showed differences in their microbial composition wherein TWAT was dominated by *Proteobacteria* followed by *Bacteroidetes, Actinobacteria* and *Cyanobacteria*, while the microbial composition of PWAT was dominated by *Actinobacteria* followed by *Bacteroidetes, Proteobacteria, Planctomycetes* and *Cyanobacteria*. Interestingly, OTUs belonging to phylum *Nitrospirae*, *Chloroflexi*, *Chlamydiae* and *Acidobacteria*, were present only in the PSPO, whereas OTUs belonging to the phylum *Firmicutes* were present only in TSPO sample.

We have used SIMPER analysis to determine the OTUs (at deeper taxonomic level) contributing to the differences in TSPO and PSPO samples (Table 2). TSPO sample showed high abundance of the sequences belonging to the genus *Clostridium* (50.3 %) albeit its absence in the surrounding water sample (Table 2). *Synechococcus* (8.67 %) was the second most dominant OTU in the TSPO samples, while in PSPO samples these OTUs were absent. OTUs belonging to the *Acinetobacter, Vogesella,* and *Rhizobiales* were present only in TSPO samples. PSPO samples were mainly dominated by OTUs belonging to *Phycisphaerales*, Unclassified *Alphaproteobacteria*, *Planktothrix*, *Solirubrobacterales*, *Pirellulaceae*, *Actinomycetales*, *Rhodospirillaceae*, *Rhodocyclaceae*, etc. Interestingly, most of the OTUs present in the PSPO samples were either absent or showed very little abundance in TSPO samples (Table 2).

**Table 2:** SIMPER analysis showing the principal OTUs responsible for the differences between PSPO and TSPO samples

No.	OTUs	Dissimilarity contributions (%)	Abundance in PSPO (%)	Abundance in TSPO (%)
1	*Clostridium*	32.5	0	50.3
2	Alphaproteobacteria	7.899	12.3	0
3	*Phycisphaerales*	7.1	13	2
4	*Planktothrix*	6.453	10	0
5	*Synechococcus*	5.596	0	8.67
6	*Aeromonadaceae*	3.874	0	6
7	*Solirubrobacterales*	3.01	4.67	0
8	*Planktothricoides*	2.583	4	0
9	*Rhodospirillaceae*	1.961	3	0
10	*Actinomycetales*	2.1	4.6	1.2
11	*Acinetobacter*	1.723	0	2.67
12	*Pirellulaceae*	1.715	3.67	1
13	*Stramenopiles*	1.51	0.667	3
14	*Rhodocyclaceae*	1.51	2.33	0
15	*Flavobacterium*	1.079	1.67	0
16	Gammaproteobacteria	1.076	0	1.67
17	*Planctomyces*	1.7	2.67	0
18	*Rheinheimera*	0.8744	1.33	0
19	*Rhodobacter*	0.8667	1.33	0
20	*Comamonadaceae*	0.6457	1	2
21	*Parachlamydiaceae*	0.6457	1	0
22	*Microcystis*	0.6457	1	0
23	*Rhizobiales*	0.6457	0	1
24	*Fluviicola*	0.6457	1	0
25	*Vogesella*	0.6457	0	1

**Fig. 2:**
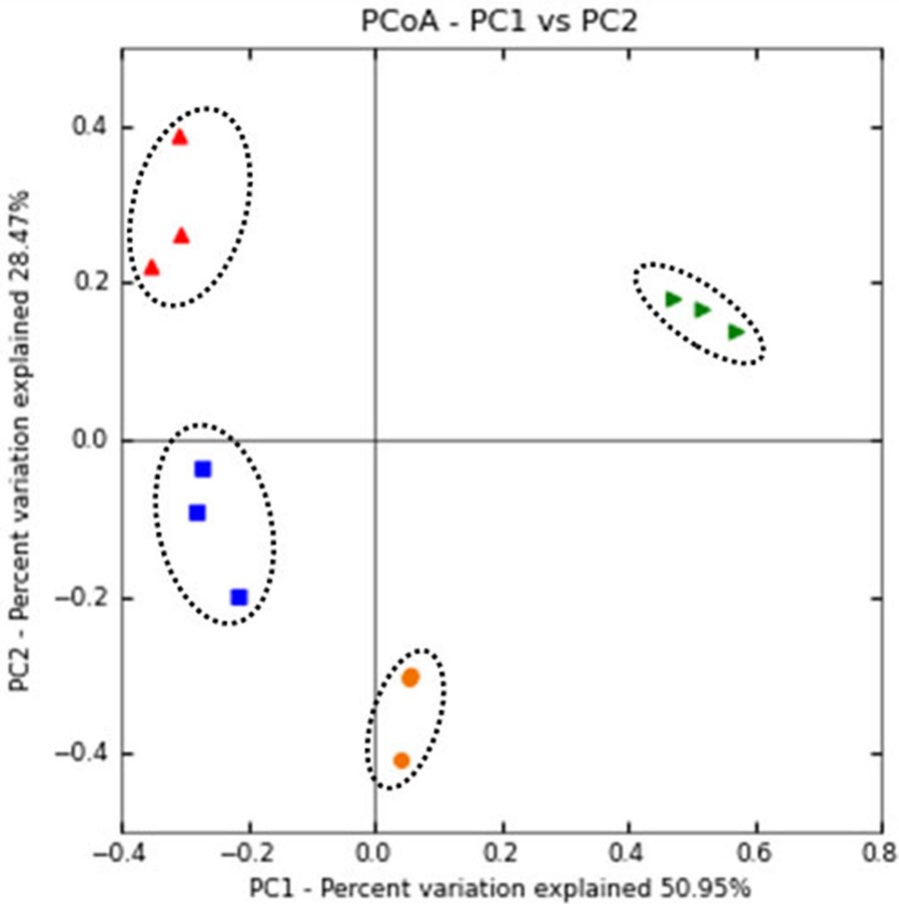
PCoA plot based on weighted unifrac distances of microbial community of samples. Microbial community of samples belonging to the same geographic locality and origin clustered together, while microbial community of samples from different geographic location formed a distinct cluster. There was distinct difference in the microbiota of two species of sponges, while difference was also observed in the microbiota of the sponges and its respective water samples. *Red triangle*: PSPO, *blue square*: PWAT, *orange circles*: TSPO and *green triangles*: TWAT

**Fig. 3:**
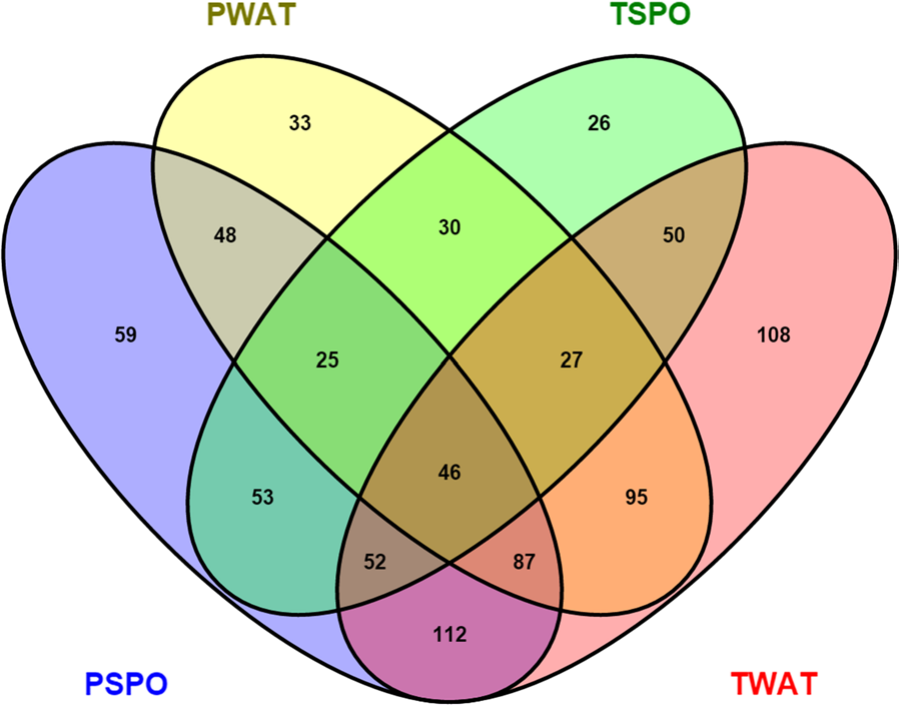
Venn diagrams of unique and shared OTUs (at genus level) of the samples studied here. Average OTU abundances of three samples belonging to respective water and sponge sample was shown here

The difference in the microbial community structure of the samples was further observed using PCoA plot based on the weighted unifrac distances (Fig. 2). Among all samples, all the replicates belonging to respective samples clustered together. PSPO samples formed distinct clade and showed notable differences from its respective water samples (PWAT). Similarly, all the individuals belonging to TSPO samples formed a distinct clade and separated by its respective water samples (TWAT). Furthermore, both TSPO and PSPO samples showed significant differences from each other which was also evident from Venn diagram that showed only 176 OTUs shared between TSPO and PSPO samples. While both TSPO and PSPO samples, shared only 175 and 206 OTUS, respectively with their respective water samples (Fig. 3).

The comparisons of microbial community structure of freshwater sponges and marine sponges (Additional file 1: Fig. S2) revealed that presence of many bacterial phyla (such as *Chloroflexi, Acidobacteria, Nitrospirae, Poribacteria, Spirochaetes, Gemmatimonadetes, Proteobacteria*) consistently in marine water sponges. While other bacterial phyla (such as *Firmicutes, Cyanobacteria, Verrucomicrobia, Actinobacteria, Chlorobi, Chlamydiae,* Candidate division *TM7, Bacteroidetes, Planctomycetes*) showed high abundances only in the fresh water sponges.

## Discussion

Compared to marine sponges, information on the microbial diversity, composition, host-microbiota interactions and the roles of their symbiont, etc. of the freshwater sponges are rare. The microbial diversity of two fresh water sponges (*E. carteri* and *C. lapidosa*) is explored for the first time using NGS technology. The present study demonstrated that like marine sponges, the microbial community structure of freshwater sponges is also highly diverse. Our study also provided compelling evidences for the distinct microbial composition of freshwater and marine sponges.

In the past, knowledge about the microbiota of fresh water sponges was limited to few bacteria (Keller-Costa et al. 2014) and it was also thought that the microbial diversity of fresh water sponges is lower compared to marine sponges (Taylor et al. 2007). Gladkikh et al. (2014) for the first time used NGS technology for the exploration of microbial diversity of freshwater sponges and identified more than 400 OTUs. Interestingly, in the present study, we identified higher microbial diversity in the freshwater sponges with OTUs ranging from 930 to 3218 (Table 1). Although OTUs obtained in the present study were comparable with OTUs obtained in marine sponges showing 1099–2996 OTUs (Webster et al. 2010), other studies on the marine sponge showed low numbers of OTUs. For example: studies on the marine sponges from the Atlantic coast, obtained only 686 OTUs (Alex and Antunes 2015), microbial diversity of marine sponges collected from different ocean obtained no more than 364 OTUs per species (Schmitt et al. 2012), and Cuvelier at el. (2014) obtained only 639 OTUs in the marine sponge, *Cinachyrella.* Thus, this study, for the first time provided evidence for the higher microbial diversity in freshwater sponges. There are several factors contributing to the high microbial diversity in the sponges; that includes, the gradual colonisation of microbes over millions of years, functional redundancy in the sponge microbiome and continuous exposure to the surrounding water (Hentschel et al. 2012). The external environment is very often considered as a seed bank for the colonisation of the microbes in the sponges. Wang et al. (2012) pointed out that the freshwater sediments harbours a higher microbial diversity compared to intertidal and marine sediments. Thus, the highly diverse external environment could also be one of the reasons for the higher microbial diversity in freshwater sponges.

Our results also revealed a significant difference in the OTUs of the both sponges (p = 0.009) and among them sample PSPO showed highest alpha diversity. We did not find the significant (p = 0.2) difference in the microbial diversity of the PSPO samples and its surrounding water. On the other hand, microbial diversity of TSPO samples and its surrounding water showed significant (p = 0.0002) difference. In fact, this is in agreement with the studies done by others that showed less diverse microbial diversity of sponges compared to its surrounding water (Cuvelier et al. 2014).

The difference in the microbial community structure of the sponges was more evident from the PCoA plot based on the weighted unifrac distances (Fig. 2). Our analysis showed that microbial community structure of the two sponges was significantly (ANOVA, p ≤ 0.0001) different from each other. Studies have shown that, the origin and habitat of the sponges play very important role in establishing the microbial community structure (Cleary et al. 2013). Our study also supported these observations as the physiochemical parameters measured for the habitat (water) of the two species of sponges showed significant differences (Additional file 1: Table S2) supporting the possible role played by habitat in establishing the microbial community structure. PCoA plot also showed that the microbiota of the sponges was significantly different from their respective water samples. Additionally, Venn diagram analysis of OTUs showed that, the sponges harboured a large number of unique OTUs compared to each other and its respective water samples (Fig. 3). The distinct microbial community structure of the sponges, compared to its surrounding water samples also implicates the influence of the host genetic factors in structuring the microbial community structure of the sponges (Costa et al. 2012).

Gladkikh et al. (2014) identified more than 24 bacterial phyla from two freshwater sponges (*L. baicalensis* and *Baikalospongia* sp.) with a predominance of *Bacteroidetes, Proteobacteria* and *Actinobacteria*. In the present study, we have identified 14 bacterial phyla in which *Proteobacteria, Firmicutes, Cyanobacteria, Actinobacteria, Planctomyces* and *Bacteroidetes* were most abundant (Fig. 1). *Firmicutes* is the most commonly found phyla in the marine sponges (Taylor et al. 2007), while in the fresh water sponges it is found in low abundance (Gladkikh et al. 2014). We reported a high abundance of *Firmicutes* in *E. carteri*, for the first time. Apart from this, although in low abundance, our study also identified OTUs belonging to phylum *Nitrospirae,Verrucomicrobia*, *Chloroflexi*, *Chlorobi, TM7, OD1* and *Chlamydiae*. The presence of these low abundant phyla also signifies the importance of NGS technology for the detection of such rare OTUs which otherwise would not be possible using traditional techniques (Lee et al. 2010). The present study also showed that microbial community structure (at the phylum level) of the fresh water sponges is significantly (ANOVA, p ≤ 0.0001) different from the marine sponges (Additional file 1: Fig. S2). The microbiota of the marine-water sponges was mainly characterised by the conspicuous presence of *Poribacteria, Gemmatimonadetes,* Candidate division *SBR1093, Spirochaetes.*

At the deeper taxonomic level, more than 50 % of the sequences from TSPO samples showed similarity with OTUs belonging genus *Clostridium*, albeit its absence in the surrounding water (Table 2). It is worth mentioning that sponge extracellular matrix is rich in proteoglycans, glycoproteins, collagen, spongin, etc. It is thought that the high abundance of *Clostridium* could also be due to their ability to modify or utilize such extracellular matrix (Hentschel et al. 2012). Interestingly, many members of the genus *Clostridium*, are also known for their fermentation capacity and antibacterial properties (Szymanowska-Powalowska et al. 2014). However, on the contrary, studies on the marine sponges have also shown the increase in the abundance of *Clostridium* due to environmental stresses or toxic chemicals (Tian et al. 2014, 2015). In this regard, further studies are needed to understand the role of *Clostridium* in the fresh water sponge, as they are also exposed to many environmental stresses.

Both marine and freshwater sponges are well- endowed with *Cyanobacteria*, mainly helping them in nitrogen fixation and protection against UV radiations (Taylor et al. 2007; Webster and Taylor 2012). Previously, *Cyanobacteria* were either undetected (Costa et al. 2012) or identified as one of the minor phyla (Gladkikh et al. 2014) in the freshwater sponges. Our study showed high abundance of *Cyanobacteria* in both the sponges, which is also comparable with the abundance of *Cyanobacteria* found in marine sponges (Cuvelier et al. 2014). Within this phylum, OTUs belonging to *Synechococcus* were abundantly present only in *E. carteri*. *Synechococcus* is one of widely spread Cyanobacterial species found in marine sponges (Cleary et al. 2013; Burgsdorf et al. 2015) and very recently their association with freshwater sponges was shown by (Gladkikh et al. 2014; Kulakova et al. 2014). In *C. lapidosa,* cyanobacterial lineages such as, *Planktothrix*, and *Planktothricoides* (20–22 %,) were present. These OTUs are commonly found in various fresh water lakes and also known for their toxicity and bloom formation (Suda et al. 2002; Penn et al. 2014). Nevertheless, the role of *Cyanobacteria* as a food or symbiont remains uncertain.

*Actinobacteria* isolated from sponges are one of the important sources of bioactive compounds and are extensively studied in marine sponges (Santos-Gandelman et al. 2014; Sun et al. 2015). In the present study, OTUs belonging to *Actinobacteria* were present in both the sponges with comparatively high abundance in PSPO samples. It is interesting to point out that the abundance of *Actinobacteria* is significantly higher in water samples but low in sponges (Fig. 1), again suggesting the ability of sponges to selectively acquire the microbes from the surroundings. Within this phylum, OTUs belonging to *Actinomycetales* and *Solirubrobacterale* were present in high abundance. Members of order *Actinomycetales* are known to show protease, antimicrobial (Li and Liu 2006), and immuno-regulatory activities (Tabares et al. 2011). Few of them are also known to produce bio surfactants (Gandhimathi et al. 2009) and bioactive natural products with potential for drug discovery (Abdelmohsen et al. 2014). However, *Solirubrobacterale* is recently described order, for which very little information is available (Williams et al. 2014). Compared to marine sponges, studies exploring such activities of *Actinobacteria* from the fresh water sponges are lacking. Our study provided evidences for the presence of of *Actinobacteria* in fresh water sponges. Future studies exploring the bioactive potential of these bacteria will shed more light on the ability fresh water sponges to produce biologically active compounds which is neglected so far.

*Planctomycetes* is one of the abundantly present phylum in the marine as well as fresh water sponges (Gladkikh et al. 2014; Alex and Antunes 2015). In this study there was a significant difference in the abundance of the *Planctomycetes* in both the samples with high abundance in PSPO. The members of this phylum, such as *Planctomyces* and *Pirellula* are known to be involved in the ammonium oxidation (Mohamed et al. 2010). More importantly, these bacteria are also considered as one of the alternative sources for waste water treatment plants (Gao and Tao 2011). The presence of these bacterial lineages in the freshwater sponges, necessitates further studies for its biotechnological interest.

*Proteobacteria* is one of the dominant phylum found in the marine sponges (Webster et al. 2010; Cleary et al. 2013). Our study is in accordance with these studies showing a high abundance of phylum *Proteobacteria* (22–39 %) in both the freshwater sponges (Fig. 1). Within this phylum, OTUs belonging to unclassified *Alphaproteobacteria* were found only in PSPO sample. Interestingly, such unclassified alpha *Proteobacteria* have also been reported from the other sponges (Cuvelier et al. 2014). Apart from this, other proteobacterial lineages such as *Aeromonadaceae, Rhodospirillaceae, Comamonadacae, Acinetobacter, Rhodocyclaceae, Gammaproteobacteria, Rheinheimera, Rhodobacter, Methylocaldum, Vogesella*, and *Rhizobiales* also showed differential abundances between two sponges studied here. Members of these bacterial lineages isolated from sponges (especially, marine sponges) are known to show antimicrobial activities and also known to produce bioactive compounds (Althoff et al. 1998; Taylor et al. 2007; Kennedy et al. 2008; Wen-Ming et al. 2010; Schmitt et al. 2012; Graca et al. 2013; Fuerst 2014). Many Gamma-proteobacterial lineages from the fresh water sponges are also known posses genes for chitinolytic enzymes (Cretoiu et al. 2012). However, studies exploring the biotechnological potential of these taxa isolated from fresh water sponges are lacking.

Very recently, Suzuki et al. (2015) suggested the exploration of novel sources, for the search of biotechnological important bacteria. For example: Lichens, as they are known to show microbes-host interaction and microbe- microbes interactions leading to production of microbial bioactive compounds. In this context, sponges are ideal sources as they harbour symbiotic bacteria. Although, freshwater sponges are not as popular as marine sponges for its ability to produce biologically active compounds, our study showed that they harbour bacteria that have a wider biotechnological potential. The present study demonstrated that even fresh water sponges harboured high microbial diversity like marine sponges. This study provided valuable information about the microbial composition and diversity of the freshwater sponges as they posses indigenous bacteria compared to its surrounding environments. This would also help us to advance our understanding of host microbiota interaction in fresh water sponges. Till now microbiota of fresh water sponges is never been explored for their biotechnological potential. This study provided the inventory of such microbes and thus, it is imperative to isolate and characterise microbes from freshwater sponges for their ability to produce compounds of biotechnological significance.

## Additional file

10.1186/s13568-016-0211-2 Collection details of the samples studied

## Abbreviations

PSPO: sponge collected from Pashan lake
PWAT: water collected from Pashan lake
TSPO: sponge collected from Talegaon lake
TWAT: water collected from Talegaon lake
