# Supplementary material for: Microbial community structure of two freshwater sponges using Illumina MiSeq sequencing revealed high microbial diversity
Source: AMB Express. 2016 Jun 14;6:40. doi: 10.1186/s13568-016-0211-2 (PMC4908081; doi:10.1186/s13568-016-0211-2)

Journal Name: AMB Express

Title: Microbial community structure of two freshwater sponges using Illumina MiSeq sequencing revealed high microbial diversity.

Swapnil Sopan Gaikwad[1,2] Yogesh S. Shouche[2], Wasudeo N. Gade[1*]

[1]Department of Biotechnology, Savitribai Phule Pune University, Pune-411007, Maharashtra, India.

[2]Microbial culture collection (MCC), First floor, Central Tower, Sai Trinity Building Garware Circle, Sutarwadi, Pashan Pune, Maharashtra 411021, India.

* Corresponding author

Dr. W. N. Gade

Professor,

Department of Biotechnology,

Savitribai Phule Pune University,

Ganeshkhind, Pune-411007.

Maharahstra, India.

Email: wngade@unipune.ac.in

Phone: +91-020-25694952.

Table S1: Collection details of the samples studied.

No.	Sample code	Locality	latitude	longitude	Collection date
1	PSPO1	Pashan, Pune, India	18.5337	73.7900	2015-01-06
2	PSPO2	Pashan, Pune, India	18.5337	73.7900	2015-01-06
3	PSPO3	Pashan, Pune, India	18.5337	73.7900	2015-01-06
4	PWAT1	Pashan, Pune, India	18.5337	73.7900	2015-01-06
5	PWAT2	Pashan, Pune, India	18.5337	73.7900	2015-01-06
6	PWAT3	Pashan, Pune, India	18.5337	73.7900	2015-01-06
7	TSPO 1	Talegaon Dabhade, Pune, India	18.7165	73.6864	2015-01-07
8	TSPO 2	Talegaon Dabhade, Pune, India	18.7176	73.6896	2015-01-07
9	TSPO 3	Talegaon Dabhade, Pune, India	18.7174	73.6899	2015-01-07
10	TWAT 1	Talegaon Dabhade, Pune, India	18.7165	73.6864	2015-01-07
11	TWAT 2	Talegaon Dabhade, Pune, India	18.7176	73.6896	2015-01-07
12	TWAT 3	Talegaon Dabhade, Pune, India	18.7174	73.6899	2015-01-07

Table S2: Ecological parameters measured for the water samples collected from the two habitats. The measurements were done in triplicates, mean value are shown here and standard error in parenthesis. P - value are calculated using T test in PAST software.

No	Ecological parameters	TWAT	PWAT	p value
1	DO (mg/L)	6	4.5	< 0.0001
2	TDS (mg/L)	240 ± 0.88	255 ± 2.9	0.01
3	Nitrite (mg/L)	0	3.13 ± 0.06	0.0002
4	pH	7.1	8.2 ± 0.033	< 0.0001
5	Phosphate (mg/L)	1.86 ± 0.033	1	< 0.0001
6	Ammonia (mg/L)	0.66 ± 0.033	0.8	< 0.0001
7	Temperature (°C)	23	27	< 0.0001

Fig S1: The distribution of bacterial community between TSPO and PSPO samples. Bacterial abundance in the PSPO sample has a positive difference between proportions (blue circles), whereas bacterial abundance in the TSPO sample has a negative difference between proportions (green circles). Bars on the left represent the proportion of each bacterial phyla abundance in the samples. p values are shown in the right side.

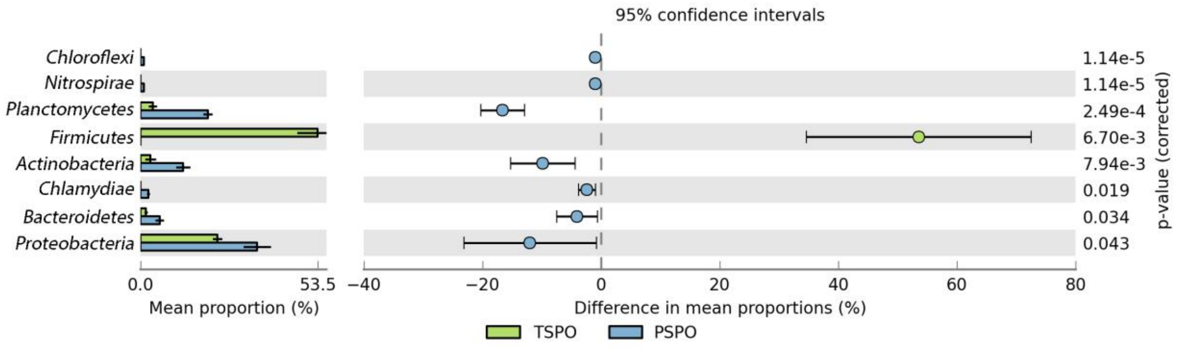

Fig S2: Two dimensional non-metric multidimensional scaling (NMDS) of the bacterial communities of the freshwater sponges and marine sponges. A convex hull encloses all samples of the same category forming distinct cluster. NMDS plot is based on the Bray-curtis similarity index. There was significant difference in microbial community structure of marine sponges and freshwater sponges.

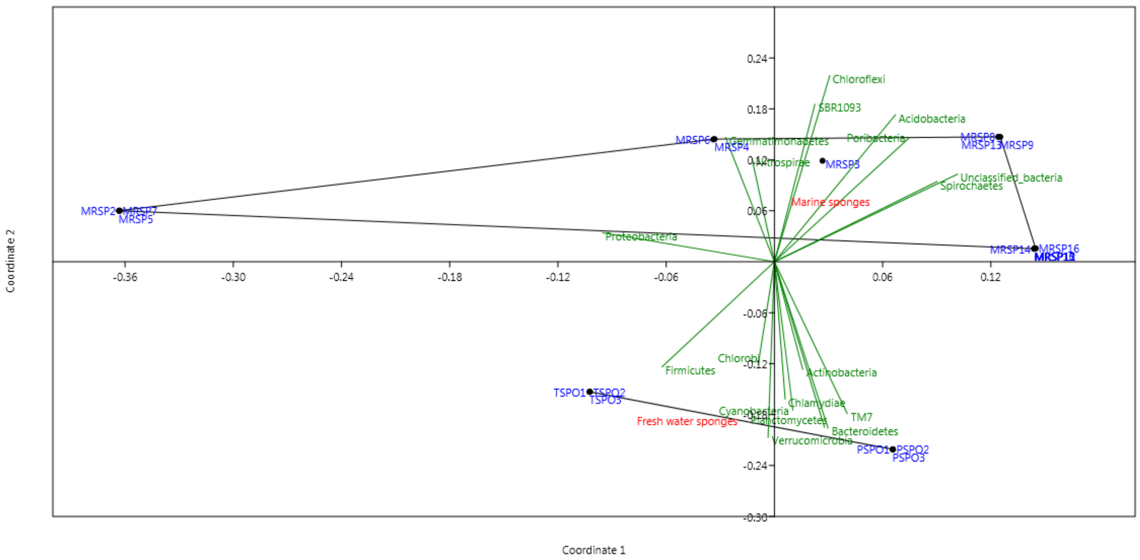

Supplement: Supplementary file 1 — 10.1186/s13568-016-0211-2 Collection details of the samples studied [file 13568_2016_211_MOESM1_ESM.pdf]
